# Molecular insights into the structure and function of the *Staphylococcus aureus* fatty acid kinase

**DOI:** 10.1016/j.jbc.2024.107920

**Published:** 2024-10-24

**Authors:** Megan J. Myers, Zhen Xu, Benjamin J. Ryan, Zachary R. DeMars, Miranda J. Ridder, David K. Johnson, Christina N. Krute, Tony S. Flynn, Maithri M. Kashipathy, Kevin P. Battaile, Nicholas Schnicker, Scott Lovell, Bret D. Freudenthal, Jeffrey L. Bose

**Affiliations:** 1 Department of Microbiology, Molecular Genetics and Immunology, University of Kansas Medical Center, Kansas City, Kansas, USA; 2 Protein and Crystallography Facility, University of Iowa, Iowa City, Iowa, USA; 3 Department of Biochemistry and Molecular Biology, University of Kansas Medical Center, Kansas City, Kansas, USA; 4 Computational Chemical Biology Core, University of Kansas, Lawrence, Kansas, USA; 5 Protein Structure & X-Ray Crystallography Laboratory, University of Kansas, Lawrence, Kansas, USA; 6 NYX, New York Structural Biology Center, Upton, New York, USA

**Keywords:** MRSA, *Staphylococcus aureus* (*S. aureus*), proline isomerization, fatty acid metabolism, fatty acid binding protein, protein-protein interaction, membrane, fatty acid kinase, kinase

## Abstract

Gram-positive bacteria utilize a Fatty Acid Kinase (FAK) complex to harvest fatty acids from the environment. This complex consists of the fatty acid kinase, FakA, and an acyl carrier protein, FakB, and is known to impact virulence and disease outcomes. Despite some recent studies, there remain many outstanding questions as to the enzymatic mechanism and structure of FAK. To better address this knowledge gap, we used a combination of modeling, biochemical, and cell-based approaches to build on prior proposed models and identify critical details of FAK activity. Using bio-layer interferometry, we demonstrated nanomolar affinity between FakA and FakB which also indicates that FakA is dimer when binding FakB. Additionally, targeted mutagenesis of the FakA Middle domain demonstrates it possesses a metal binding pocket that is critical for FakA dimer stability and FAK function *in vitro* and *in vivo*. Lastly, we solved structures of the apo and ligand-bound FakA kinase domain to capture the molecular changes in the protein following ATP binding and hydrolysis. Together, these data provide critical insight into the structure and function of the FAK complex which is essential for understanding its mechanism.

*Staphylococcus aureus* is a Gram-positive bacterium asymptomatically colonizing up to 30% of the general population and an even higher percentage in infants (1, 2). In addition, it is the leading cause of skin infections and can cause a myriad of diseases with high morbidity and mortality in both the nosocomial and community environments (3, 4, 5, 6). Indeed, it is a global health problem estimated to kill one million people worldwide annually (7). This is due to the plethora of virulence factors produced by the bacterium, its metabolic diversity to adapt to various niches, and the high frequency of antibiotic resistance (8, 9, 10, 11, 12, 13, 14, 15, 16, 17). New options to combat *S. aureus* infections are critically needed, which requires a more detailed understanding of the physiology, pathogenesis, and virulence of this pathogen. Due to differences between bacterial and host pathways, fatty acid metabolism and biosynthesis are attractive targets for novel therapeutic development.

Fatty acids are an essential component of life as they are the building blocks of membranes. They are common to the bacterial environment during infection as part of host cells, as a component of low-density lipoproteins, and as free fatty acids. This is particularly relevant to skin and soft tissue infections as host saturated and unsaturated fatty acids (UFAs) are ample, with the latter known to be antimicrobial (18, 19, 20). Because of the abundance of fatty acids within the host environment, bacteria have adopted ways to acquire exogenous fatty acids (exoFAs). ExoFAs can serve to supplement bacterial endogenous fatty acid biosynthesis by the FASII system or be degraded by β-oxidation. Because of this, exoFA acquisition contributes to resistance to FASII inhibitors, such as Triclosan (21, 22, 23, 24). As discovered in *S. aureus*, Gram-positive bacteria can acquire exoFAs using the Fatty Acid Kinase (FAK) complex which imports and activates exoFAs to be primarily inserted into lipids (9, 10). The FAK complex consists of FakA, an ATP kinase, and FakB, an acyl carrier protein that brings the fatty acid substrate to FakA for phosphorylation (25). *S. aureus* contains two FakB proteins. FakB1 prefers saturated fatty acids and has been proposed to scavenge saturated exoFAs or recycle those made or released by *S. aureus* (26, 27). FakB2 preferentially interacts with UFAs and thus binds host fatty acids since *S. aureus* does not synthesize UFAs (27, 28). In this process, exoFAs passively diffuse into the bacterial membrane and are bound by FakB to be brought into the cytosol (26). By a poorly understood mechanism, FakB interacts with FakA to form the FAK complex, whereby the kinase activity of FakA phosphorylates the fatty acid. This is processed by the phospholipid synthesis protein PlsY to insert the fatty acid primarily into the *sn1* stereospecific position of phospholipids (29). Without the FAK complex, *S. aureus* is unable to utilize exoFAs to synthesize lipids (28, 29).

FakA was originally discovered due to its impact on *S. aureus* virulence factor production and was termed VfrB; since then, it was determined to be a fatty acid kinase and renamed FakA (9). The FAK complex is important for a variety of *S. aureus* cellular processes including susceptibility to host fatty acids, membrane fluidity, toxin production, and virulence depending on the host niche (9, 10, 11, 12, 13, 14, 15, 16, 17). Despite these studies demonstrating the importance of FAK in *S. aureus*, much is still not known about the functional complex. Indeed, the basic understanding of the composition of the complex and the mechanism by which fatty acid phosphorylation occurs is unknown. Previous structural studies identified key insights into the structure and activity of FakB proteins including their loose specificity for saturated and unsaturated fatty acids and the mechanism by which they remove fatty acids from the membrane (26, 27). Two additional studies provide insightful, yet conflicting models, as to the assembly and function of each domain in the Fak complex, specifically how FakA and FakB assemble into a functional complex (30, 31). Indeed, many aspects of the FakA and FakB proteins and how the complex functions have yet to be elucidated. This is highlighted by the fact that the two recent studies propose conflicting models on the FakA/B interaction (30, 31). In addition, any conformational changes that occur within FakA upon binding ATP and during catalysis are completely unknown. To address this, we used a combination of biochemical, structural, and cellular approaches to further elucidate key aspects of the functional FAK complex in *S. aureus*. We identified the importance of a Zn ion-binding pocket in the maintenance of the FakA homodimeric state and the formation of the functional FAK complex, adding clarity to the middle domain whose function was previously unclear. In addition, we identified the structural changes that occur within the FakA kinase domain throughout catalysis, including the movement of an activating loop necessary to bind ATP. Together these results provide crucial missing information about the activity of FakA, structural changes that occur within the FakA kinase domain to allow the assembly of a functional complex, and the impact of these elements on FAK complex activity.

## Results

### FakA–FakB interaction

**Figure 1:**
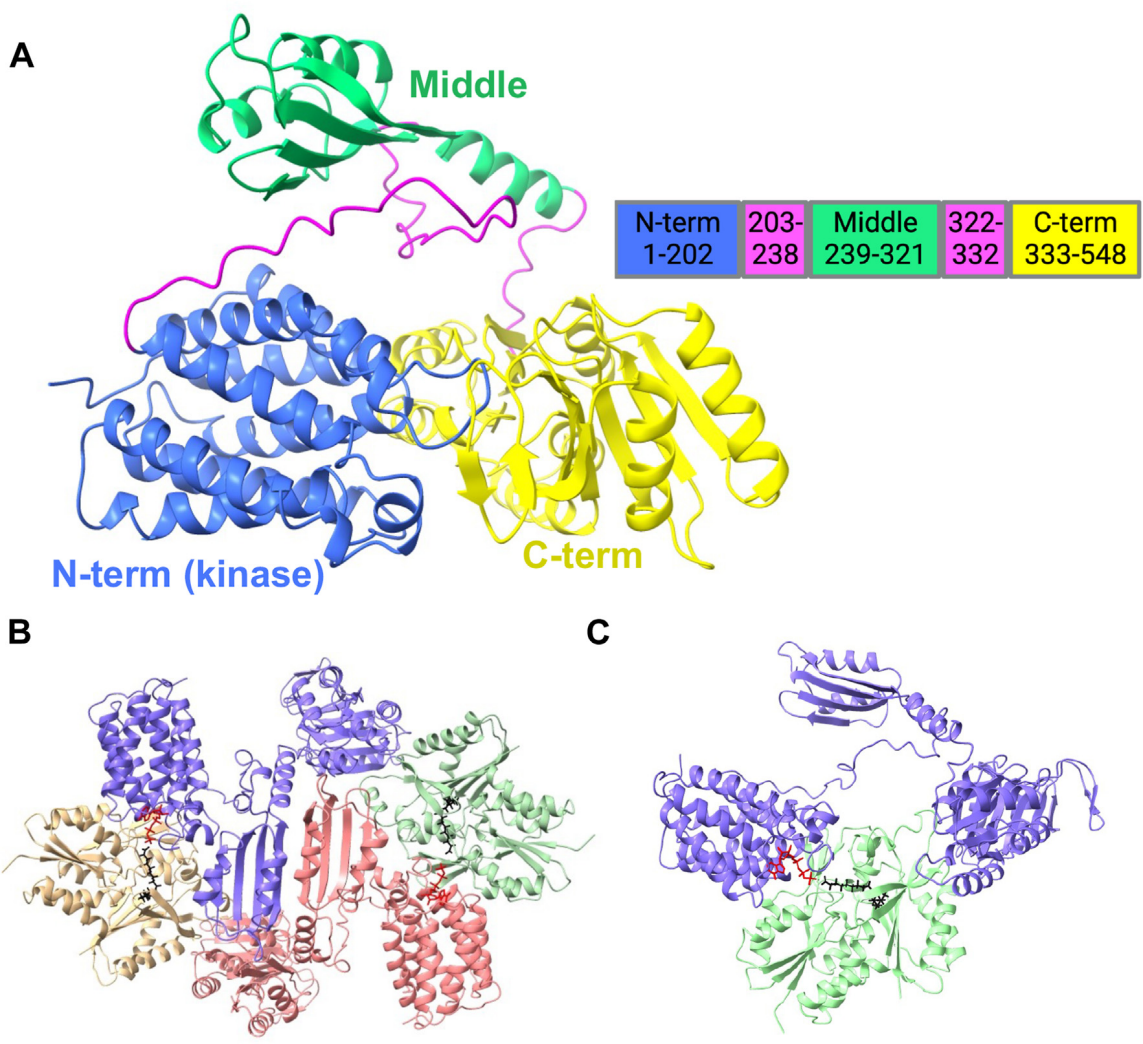
**Models of FakA FakB2 interactions.***A*, FakA AlphaFold predicted model and amino acid residues. *Blue*: N-terminal kinase domain, *Green*: Middle domain, *Yellow*: C-terminal domain, *Magenta*: unstructured regions. *B*, AlphaFold model of FakA-FakB2 heterotetramer. *C*, AlphaFold model of FakA-FakB2 heterodimer. AMP-PNP is rendered as *red cylinders* and oleic acid in *black*.

**Figure 2:**
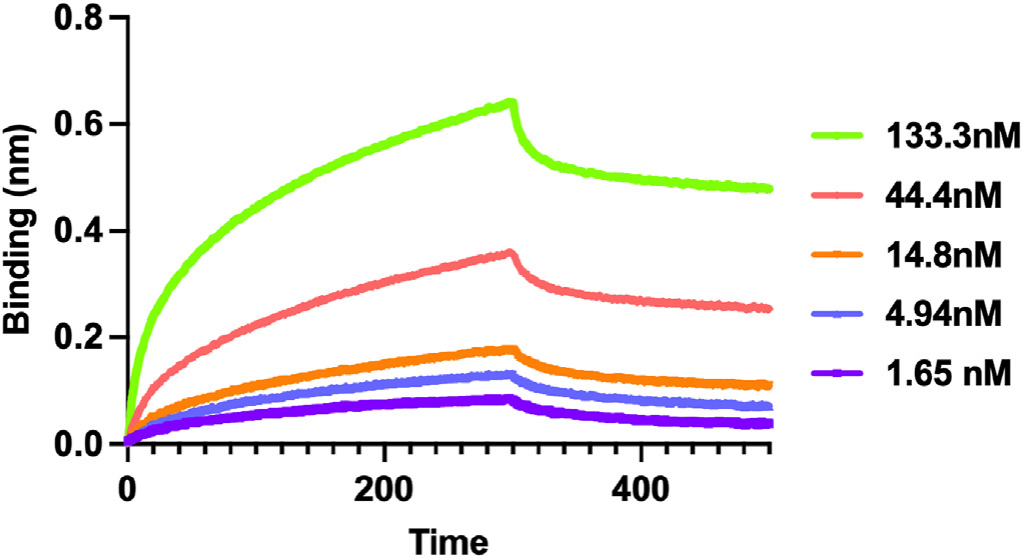
**FakA and FakB2 interact.** BLI using sequential treatment of His-FakB2, 0.1 mg·mL^−1^ BSA, buffer (wash), buffer (baseline), indicated concentration of FakA, and buffer (dissociation). Estimated K_D_ 4.34 nM (1:1 analysis) or 6.65 nM (bivalent analyte analysis (1:2). Data shown are from a representative experiment.

FakA studies have identified the three domains (Fig. 1*A*). The N-terminal domain binds ATP and is where kinase activity occurs. The Middle domain is responsible for homodimerization. The importance of the C-terminal domain has not been fully elucidated. FakA is known to interact with FakB as part of the FAK complex; however, the details of this interaction remain elusive (27, 30, 31). Recent papers pose two alternative putative models for the formation of the FAK complex. Subramanian *et al.* used *S. aureus* proteins and described a model in which the FakA N-terminal domain interacts directly with FakB1, allowing FakA to phosphorylate the FakB-bound fatty acid (30). Alternatively, Shi *et al.* assert a model using *Streptococcus suis* Fak proteins in which the N- and C-terminal domains form the active site and FakB must transfer the fatty acid to FakA by a yet unknown mechanism (31). How the complex assembles in an activated state and if FakA remains dimerized during fatty acid phosphorylation is unclear as is whether the complex formation is the same in all bacteria that possess FAK. No studies have solved the structure of an active FAK complex, *i.e.* one that contains a fatty acid. Most models for FAK propose that FakA and FakB form a higher-order structure, but this has not been defined. AlphaFold Multimer predicts a variety of potential complexes that include both single FakA to FakB interactions and others that include heterotetrameric complexes (Figs. 1, *B* and *C* and S1). Most models orientate the headgroup of the fatty acid in FakB within the proximity of the ATP in the kinase domain of FakA. To gain insight into the fundamental question of whether FakA remains as a dimer upon FakB binding, we performed bio-layer interferometry on FakA and FakB2 of *S. aureus*. Briefly, His-FakB2 was immobilized on Ni-NTA biosensor tips and then exposed to varying concentrations of FakA. The results show a strong interaction with a dissociation constant (K_D_) of ∼4.34 nM when measured as a 1:1 ratio of FakA:FakB2 (R^2^ = 0.97 and a χ^2^ = 14.68, Fig. 2 and Table S1). To investigate the complex composition, this data was compared to an alternative mathematical model in which dimerized FakA binds immobilized FakB2. Using this approach, BLI data was re-calculated using bivalent analyte computation, giving a new K_D_ of 6.65 nM (Fig. 2, and Table S1). This model fits the data more closely with an R^2^ value of 0.98 and a χ^2^ value of 2.55. This supports the hypothesis that FakA binds FakB2 with high affinity and is the first direct data supporting that FakA is a dimer upon FakB2 binding.

### FakA homodimerization is mediated by metal-bound Middle domain

**Figure 3:**
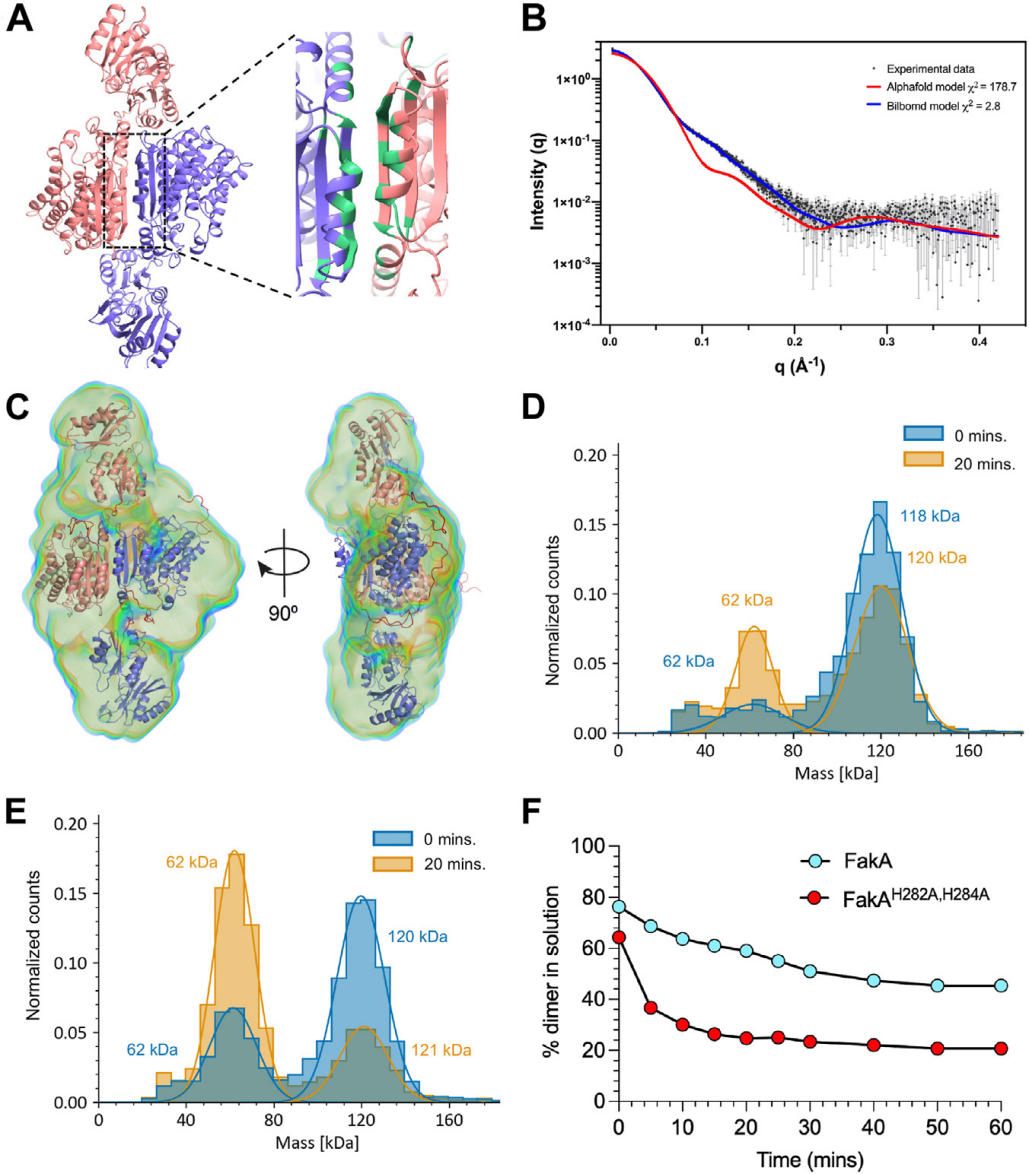
**FakA is a homodimer mediated by the Middle domain in solution.***A*, AlphaFold model of the FakA homodimer. Predicted interacting residues are shown in *green*. *B*, Crysol fitting of BilboMD models with scattering intensity (I(q)) over the radial distance (q) experimental data. *C*, molecular dynamics-based FakA model generated by BilboMD using the AlphaFold model panel (*A*) as an input overlayed within the electron density model of FakA using DENSS. The flexible regions are in *red* between rigid bodies (*Purple* and *Pink*). *D*, mass photometry of WT FakA. Measurements were taken at 0 (*blue*) and 20 min (*orange*). *E*, mass photometry of FakA^H282A, H284A^ performed as described in panel (*B*). *F*, kinetic mass photometry showing the percent of wild-type in solution that is a dimer (*light blue*) compared to percent of FakA^H282A, H284A^ present as a dimer (*red*). Data represents the mean (n = 3) with SEM. All MP experiments were performed at a final concentration of 10 nM.

Our early purification studies indicated that FakA forms a higher molecular mass than a monomer (25). More recent studies support FakA existing as a dimer but propose different points of contacts, which may result partially from the techniques used and source of FakA protein, *i.e. S. aureus versus S. suis.* To add clarity to this we sought to investigate FakA dimerization in more detail. AlphaFold-Multimer (32, 33) modeling indicates dimerization is likely to occur through specific interactions in the Middle domain (Fig. 3*A*). We sought to determine whether this is true for FakA in solution by coupling size-exclusion chromatography with small-angle X-ray scattering (SEC-SAXS) (Figs. 3, *B* and *C*, S2, Tables S2, and S3). SAXS analysis revealed that FakA exists as a dimer, with a radius of gyration (R_g_) of 42.3 Å, and a maximum dimension of 160 Å (Table S2). The corrected Porod volume and molecular weight estimated from SAXS data is also consistent with FakA forming a dimer (Table S2). To obtain a good fit of the predicted AlphaFold dimer structure required refinement with BilboMD. Specifically, flexible modeling of disordered residues at the linker regions connecting the N-terminal, Middle, and C-terminal domains (χ^2^ = 2.8; Figs. 3, *B* and *C*, S2, Tables S2, and S3). SAXS-derived molecular envelopes (bead-based models by DAMMIF and electron density models by DENSS) support the model that the FakA dimer is maintained by contacts within the Middle domain (Figs. 3*C* and S2).

We predict that FakA primarily exists as a dimer in solution but that it may or may not dissociate as a dynamic process. To investigate this, we examined dimer stability by a kinetic mass photometry assay in which purified FakA was diluted to 10 nM to trigger the dissociation of the dimer, and the ratio of monomer:dimer was tracked over time. The initial reading of FakA revealed that 77% of the protein was within a large peak at ∼118 kDa, consistent with the dimer molecular weight, as opposed to the smaller molecular mass peak of the monomer (∼62 kDa) (Fig. 3, *D* and *F*). The FakA dimer was relatively stable, reaching an equilibrium with ∼57% of the protein in the dimer peak after 20 min (Fig. 3, *D* and *F*).

**Figure 4:**
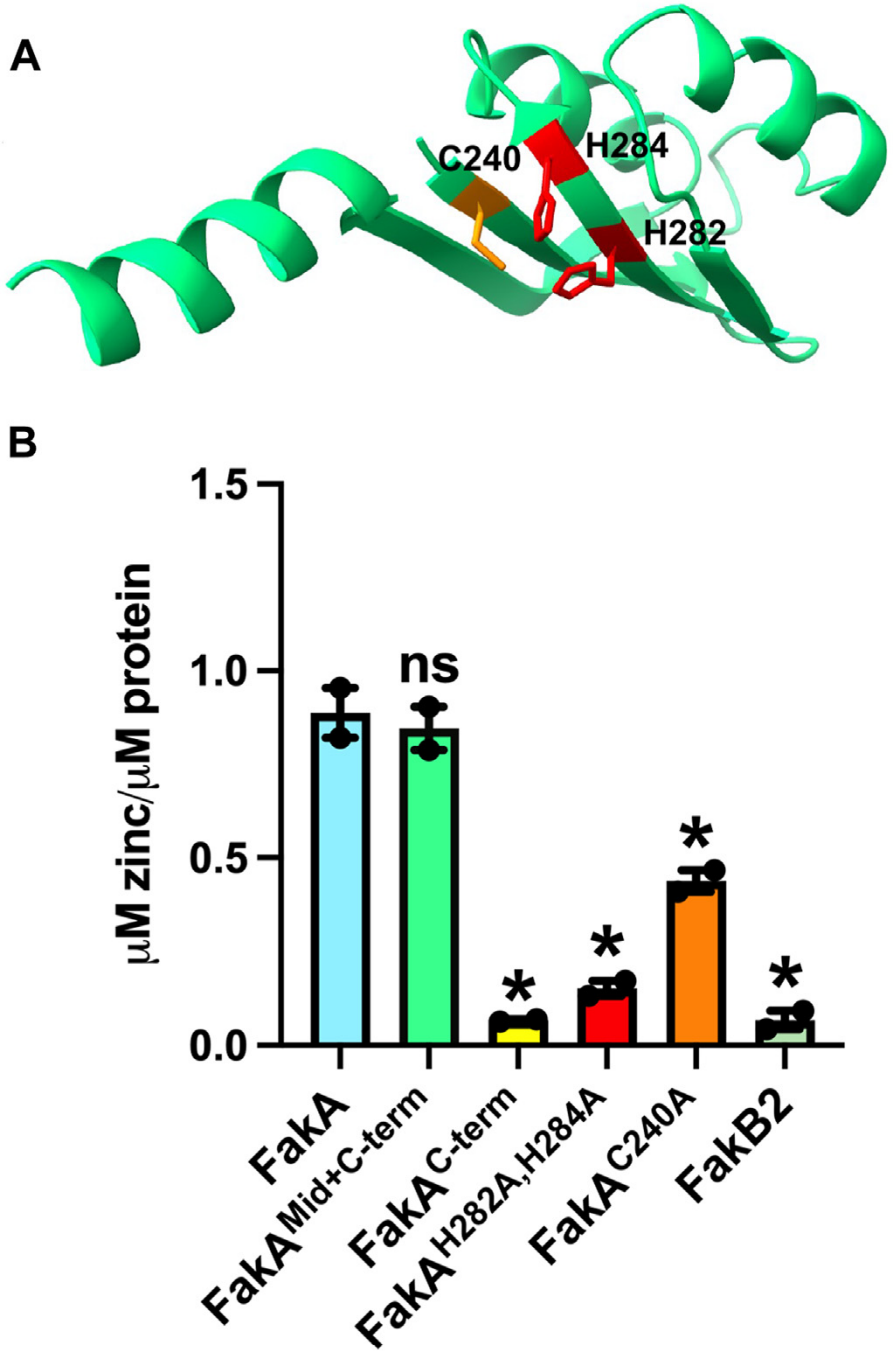
**FakA Middle domain binds zinc.***A*, AlphaFold model of Middle domain with C240, H282, and H284 shown in *orange*/*red*. *B*, ICP-MS analysis of zinc associated with the indicated proteins. Untagged FakA is used in panel (*B*). ∗ indicates *p* < 0.01 by one-way ANOVA with Dunnett *post hoc* analysis compared to full-length wild-type FakA. Bars represent the mean (n = 2) with SEM.

It is clear from our studies and others that the Middle domain of FakA plays a central role in FakA dimer formation (30, 31). However, the function of the Middle domain is uncertain as it has also been proposed to form part of the active site. This is based on results demonstrating that three conserved residues, His282, His284, and Cys240 are essential kinase activity using purified proteins (30). Our own analysis indicated that these residues form a metal-binding pocket, and we sought to validate this finding and test its importance for the FakA function (Fig. 4*A*). To investigate the presence of a metal ion, we compared purified FakA to a buffer control. In this initial screen, the greatest observed difference between the buffer control and protein samples was a 55-fold increase in the amount of zinc present in FakA (data not shown). To isolate binding to the Middle domain, we purified the C-terminal FakA with and without the Middle domain and found that only the truncated protein containing the Middle domain contained a Zn ion (Fig. 4*B*). Finally, to distinguish if the putative metal-binding pocket was responsible for Zn ion (Zn^2+^) binding, we purified a FakA^H282A, H284A^ mutant and a FakA^C240A^ mutant. Substitution of C240 with alanine led to a significant decrease in the amount of zinc ion present and this was further decreased in the FakA^H282A, H284A^ mutant (Fig. 4*B*). This is consistent with the predicted, but untested, role of the Middle domain in binding zinc in *S. suis* (31).

**Figure 5:**
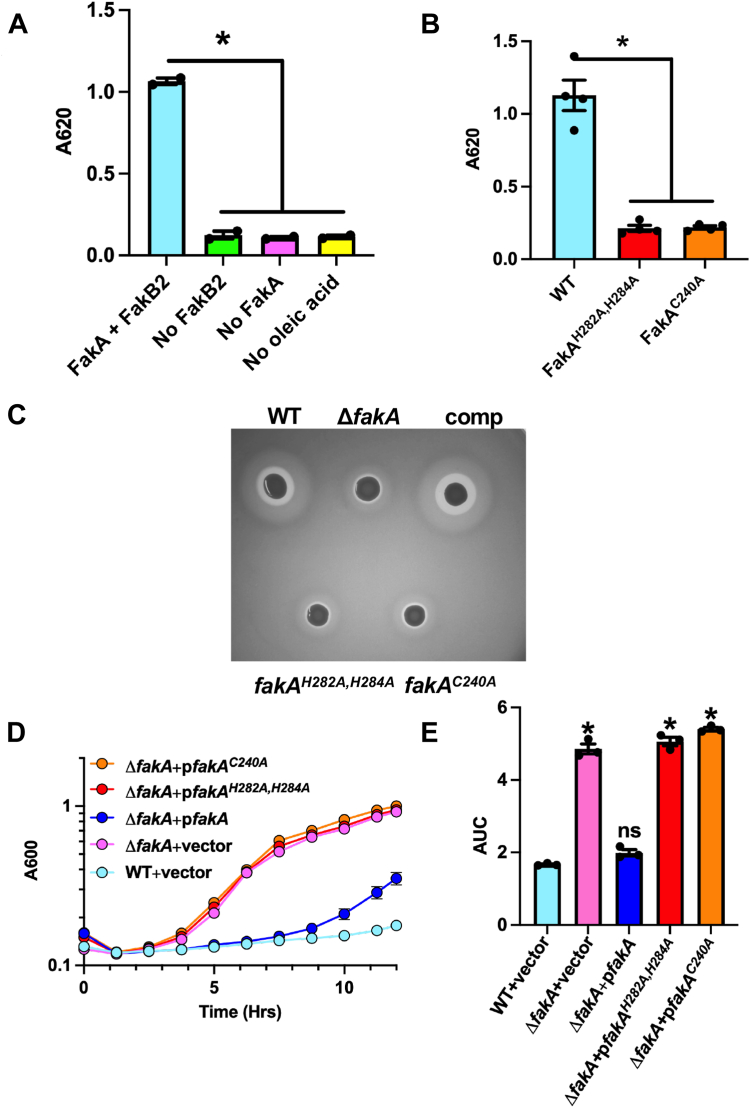
**Metal coordination within Middle domain is essential for FakA function.***A*, kinase assay for the FAK complex activity or controls of no FakB, no FakA, or no oleic acid. Proteins were His-tagged. *B*, FAK complex kinase assay indicating loss of kinase activity in variants in which Cys240 was replaced with Ala and when His282 and His284 were replaced with Ala. All proteins were purified using a GST tag which was removed prior to assay. Bars are representative of the mean [n = 2 for (*A*) or 4 for (*B*)]. *C*, rabbit blood hemolysis (clearing around colony) of WT, *fakA* mutant, or mutant with a plasmid expressing WT FakA (comp), FakA^H282A, H284A^, or FakA^C240A^. *D*, growth of variants in TSB supplemented with 192 μM (0.006%) linoleic acid. Symbols represent that mean (n = 3) with SEM. *E*, area under the curve analysis of panel (*D*). Bars represent that mean (n = 3) with SEM. Data are from a representative experiment. ∗ indicates *p*-value <0.01 by Welch’s *t* test.

Our results determined the Middle domain of purified FakA binds Zn^2+^ and we wanted to confirm the importance of this in both an enzymatic and cell-based assay. To test FakA kinase activity, we developed a malachite green kinase assay. This assay provides a simple, effective method for testing the ability of FakA to function *in vitro* using purified proteins. As expected, no activity was observed when FakA, FakB2, the fatty acid oleic acid, or ATP were omitted (Fig. 5*A*). Compared to wild-type FakA, the FakA^C240A^ and FakA^H282A, H284A^ substitutions each led to a loss of FakA kinase activity (Fig. 5*B*). While previous studies have investigated loss of function *via* alternative *in vitro* techniques, the importance of the metal binding pocket on FakA’s *in vivo* function has never been examined. Thus, we tested if our biochemical loss of these FakA mutants’ kinase activity translated to a biological difference in FakA activity in *S. aureus* using two assays previously shown to be impacted by FakA activity: α-toxin production and linoleic acid resistance (9, 13). As previously shown (11, 12), the *fakA* mutant showed a lack of α-toxin activity compared to the wild-type strain due to changes decreased activity of the SaeRS regulatory network (Fig. 5*C*). When either C240 or both H282 and H284 were replaced with Ala, hemolysis activity was not restored, demonstrating that these amino acids are critical to FakA activity (Fig. 5*C*). We then tested whether FakA^C240A^ and FakA^H282A, H284A^ would restore the sensitivity of the *fakA* mutant to linoleic acid. The linoleic acid resistance of these variants was similar to that of the *fakA* mutant (Figs. 5, *D* and *E*, and S3). Together, these data demonstrate that FakA possesses a metal binding pocket in the Middle domain that is essential to FakA kinase activity *in vitro* and FakA function *in vivo*. We predict that disruption of this metal binding pocket perturbs the structural integrity of the Middle domain, rendering the protein non-functional.

The Middle domain consists of α-helices and β-sheets, and we predicted that the metal stabilizes the β-sheets. This, in turn, would stabilize the domain, and removal of the metal would be predicted to destabilize the secondary structure and consequently dimer formation of FakA. Thus, our FakA^H282A, H284A^ protein, which does not bind Zn^2+^, would have altered dimerization capacity and dynamics. To test this, we again used a kinetic mass photometry experiment with the FakA^H282A, H284A^ mutated protein. Initially, 63% of FakA^H282A, H284A^ existed as a dimer (Fig. 3, *E* and *F*), which is less than that of wild-type FakA (Fig. 3, *D* and *F*). Equilibrium was again reached after approximately 20 min with only around 24% in the dimer form. Together, these data support a model whereby FakA forms a stable dimer in a solution that requires Zn^2+^ bound to the metal binding pocket of the Middle domain. Furthermore, it demonstrates that the metal binding pocket is critical for both FakA function *in vitro* and *in vivo*.

### FakA kinase domain undergoes structural changes during catalysis

**Figure 6:**
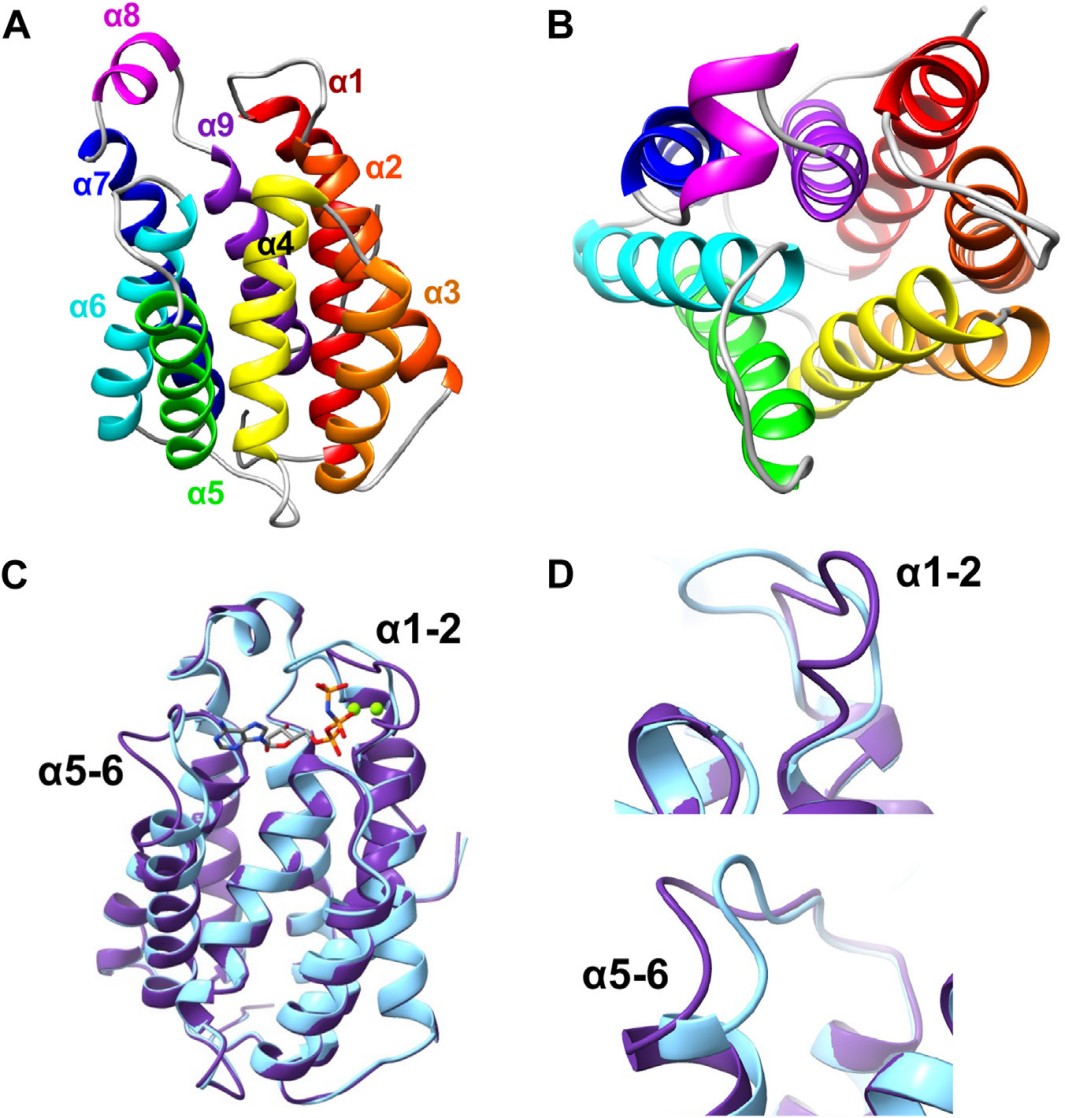
**N-terminal FakA crystal structure.***A*, Apo-FakA crystal structure with α-helices colored from N-term to C-term from *red* to *violet*. *B*, 90° rotation relative to panel (*A*) to view down α-helical barrel. *C*, overlay of Apo-FakA_N (*purple*) and AMP-PNP-FakA_N (*light blue*) with AMP-PNP labeled in *gray* and colored by element. Mg^2+^ ions are rendered as *green* spheres. Loops connecting α-helices 1 and 2 as well as five and six are labeled. *D*, zoomed in view of the α1-2 (*top*) and α5-6 (*bottom*) loop movement from apo to bound.

While our studies and others provide insight into the function of each of the three FakA domains and the composition of the FAK complex, the changes within the FakA kinase domain that occur during catalysis are unknown. To answer this, we crystallized the N-terminal domain of FakA, spanning from residue 1 to 210. To provide mechanistic insight during the catalytic cycle of FakA, we solved the structure of a novel apo form (Apo-FakA_N) and ATP analog bound form (AMP-PNP-FakA_N) as well as the previously solved ADP bound form (ADP-FakA_N). The organization of the FakA N-terminal domain in each of our structures agrees with recently solved structures (30, 31). Each of these structures formed a similar overall fold comprising a bundle of eight α-helices termed α1: G7-L31, α2: T41-N58, α3: I64-G78, α4: G81-I98, α5: S106-K122, α6: I132-N149, α7: C153-N173 and α9: S186-L202 and a short helix termed α8: A176-V181 (Fig. 6, *A* and *B*). The kinase active site sits at the top (as orientated in Fig. 6*A*) of this helical barrel near the short helix α8.

To understand the changes that occur upon ligand binding to FakA, we solved the structure of Apo-FakA_N to 1.90 Å resolution and compared it to the ligand-bound AMP-PNP-FakA_N solved to 1.15 Å resolution (Fig. 6 and Movie S1). Superposition of Apo- and AMP-PNP-FakA_N yielded an RMSD deviation of 0.48 Å between Cα atoms (201 residues) indicating relatively little difference in the overall structure of the α-helical barrel (Fig. 6*C*). However, major differences were observed in the loops connecting α1/α2 and α5/α6 (Fig. 6*D*). Upon ATP binding, the α5-6 loop moves toward the ligand binding pocket to stabilize the nucleotide. The α1/2 loop extends away from the ATP binding pocket in the apo form, then collapses to bring key residues in proximity to the γ-phosphate of ATP and to accommodate protein-protein binding during catalysis (discussed below).

**Figure 7:**
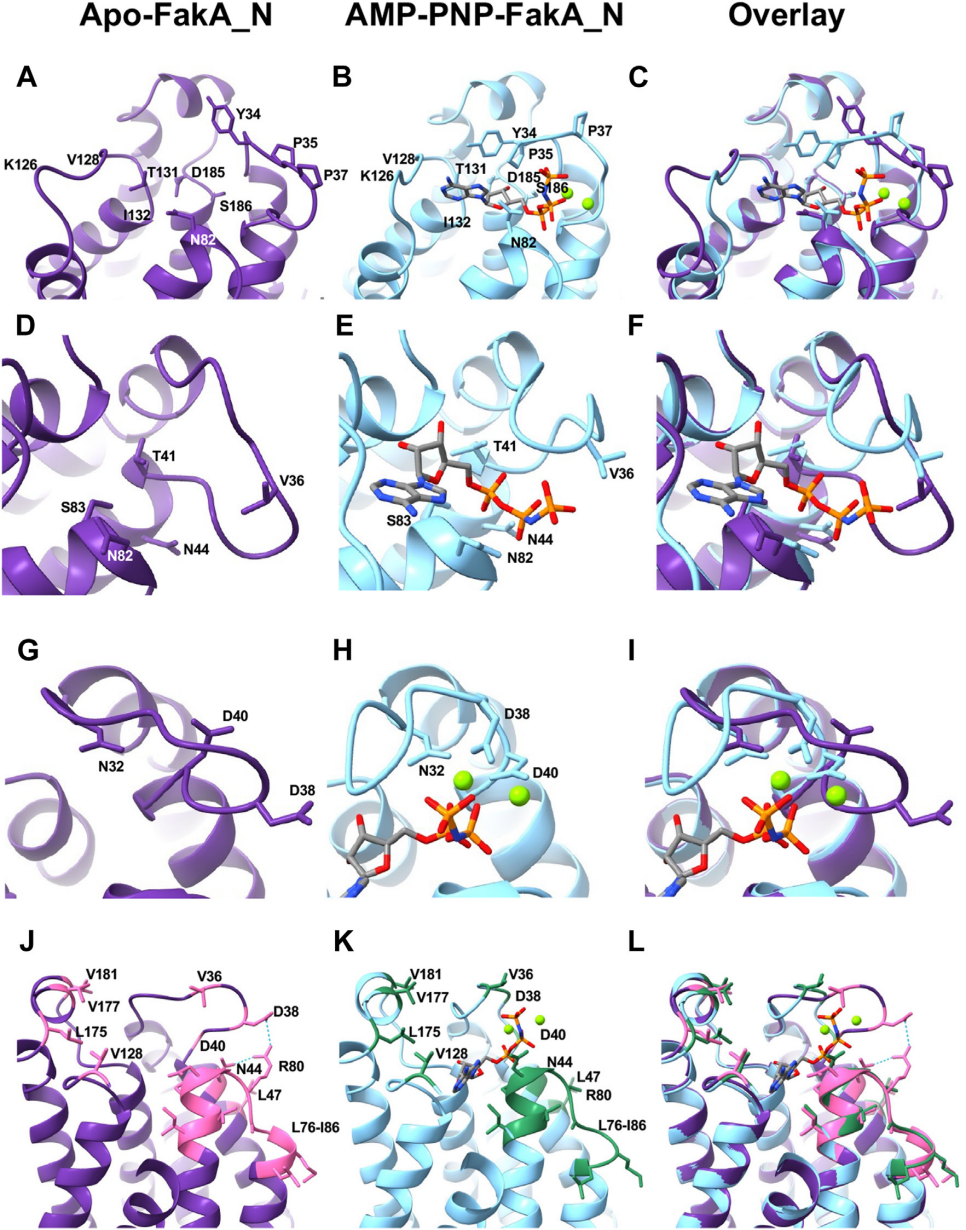
**N-terminal FakA regions of interest.***A*–*C*, nucleobase and sugar-binding pocket of FakA_N. *D*–*F*, triphosphate binding of FakA_N. *G*–*I*, Mg^2+^ coordination by FakA_N. *J*–*L*, altered binding face of FakA_N for FakB interaction. Apo-FakA_N is rendered in *purple*, and AMP-PNP-FakA_N is rendered in *blue*. Mg^2+^ ions are rendered as *green* spheres. AMP-PNP is rendered in *gray* cylinders with important atoms colored by element. In *J*–*L*, conserved hydrophobic residues are rendered in *pink* or *green* for Apo- and AMP-PNP-FakA_N, respectively.

By comparing the Apo-FakA_N to AMP-PNP-FakA_N, we observed distinct localized structural changes upon ATP binding. These changes stabilize the nucleobase, sugar, and triphosphate portion of ATP in the active state. These structural changes result in a localized change of FakA, at the ATP binding site, that generates a new hydrophobic region for protein interactions. One of these changes is a significant movement of the loop between α1-2, which we term an activating loop, consisting of residues Leu31-Thr41 (Fig. 6, *C* and *D*). Of note, Pro35 shifts 10 Å upon ATP binding and isomerizes from the *trans* to *cis* form as measured from Pro35 CG in each structure. In this conformation, Pro35 forms a stabilizing interaction with the aromatic ring of Tyr34, closing over the adenosine base of the bound ATP to form the top of the ATP binding pocket in FakA (Fig. 7, *A*–*C*, and Movie S2). The loop between α5/α6 is shifted by ∼5 Å upon binding AMP-PNP, as measured from the backbone of Lys126 (Fig. 6, *C* and *D*), to form hydrogen bonds between the backbone carbonyl of Lys126 to the N6 position, the backbone of Val128 to the N1 position, and Thr131 side chain to the N3 position of the nucleobase (Figs. 7, *A*–*C*, S4*A*, and Movie S2). This, along with a side chain conformational change and hydrogen bond in Asn82, forms the nucleobase binding pocket providing specificity for adenosine (Figs. 7, *A*–*C*, S4*A*, and Movie S2). All other residues in this α5/α6 loop region are similar in both structures. Additionally, hydrogen bonds between the sugar portion of the nucleotide and FakA_N are made between the backbone of Ile132 and O2, the side group of Asp185 with O2 and O3, and the side chain of Ser186 and O3 on the nucleotide (Figs. 7, *A*–*C*, S4*A*, and Movie S2). At the triphosphate portion of the nucleotide, the shift of the α1-2 loop creates several stabilizing hydrogen bonds through the following contacts: the backbone nitrogen of Val36 to oxygen (O1G) on the γ-phosphate, between the sidechain of Thr41 to α-phosphate oxygen-1 (O1A), the sidechain of Asn44 to the α-phosphate oxygen-2 (O2A), the side chain and backbone of Ser83 to O2A, and the backbone and sidechain of Asn82 hydrogen bonds to O1B and N3B, respectfully (Figs. 7, *D*–*F*, S4*B*, and Movie S3). Each of these hydrogen bonds provides specificity and stability to the triphosphate region of ATP.

**Figure 8:**
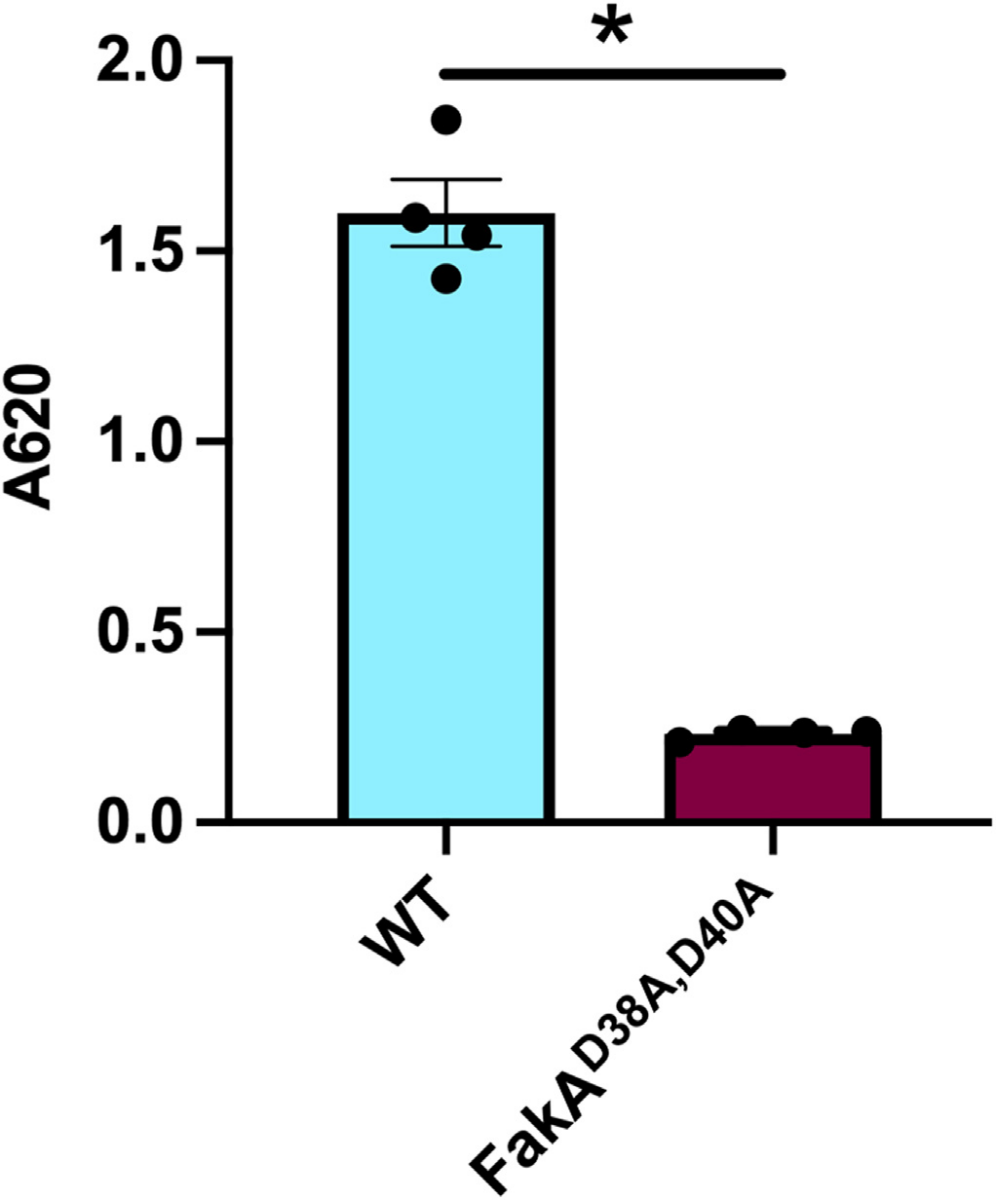
**Asp38 and Asp40 are critical for FakA kinase activity.** Kinase assay showing activity is a loss in FakA^D28A, D40A^ variant in which Asp38 and Asp40 were replaced with alanine. All proteins are His-tagged. Bars representative of the mean (n = 4) with SEM from a representative experiment. ∗ indicates *p* < 0.01 by Welch’s *t* test.

Upon ATP binding, Asp38 and Asp40 within the α1-2 loop reposition 8.25 Å and 2.81 Å, respectively to coordinate the two Mg^2+^ ions (Fig. 7, *G*–*I* and Movie S4). The two Mg^2+^ coordinate the α-, β-, γ-phosphate of the AMP-PNP as well as the catalytic triad Asp38, Asp40, and Asn32 (Fig. 7, *G*–*I* and Movie S4). To verify these amino acids are involved in kinase activity, we used our malachite green Kinase assay. In agreement with Asp38 and Asp40 being part of the catalytic site, no activity was measured when both Asp residues were substituted with Ala (Fig. 8), consistent with our prior *in vivo* work showing these residues are critical for FakA function (11).

Opposing models suggest that FakB either binds FakA in a way that brings the FakB-bound fatty acid directly to the active site on the kinase domain or FakB binds to the C-terminal domain of FakA which receives the fatty acid to be presented to the FakA N-terminal kinase domain. Both of these models would require a FakA_N-protein interaction. Comparing the Apo- and AMP-PNP-FakA_N structures also provides insight into the structural changes that would accommodate protein interactions while ligands are bound, but prevent complex formation in the absence of ATP and Mg in FakA. Upon ATP binding, the repositioning of Val36 from the interior of the protein outward after the shift in the α1-2 loop creates a new binding face that we expect to be recognized for binding and would help orient the fatty acid substrate in proximity to the γ-phosphate of ATP during FakB binding (Figs. 7, *J*–*L*, S5, and Movie S5). The movement of this activating loop exposes a group of conserved hydrophobic residues including Val181, Val177, Leu175, Val128, Leu47, and the loop between Leu76-Ile86 when FakA is bound to ATP, thus providing a new face of the protein we expect to facilitate protein-protein interactions (Figs. 7, *J*–*L*, S5, and Movie S5). In Apo-FakA_N, the α1-2 loop protrudes away from the FakA ligand binding site; we predict this prevents premature binding of the other protein partner through steric hindrance (Fig. 7, *J*–*L*). Hydrogen bonds between Asn44 and Asp38 with Arg80 stabilize this open form of FakA N-term, before ATP binding, then collapse and destabilize when in the ATP bound, closed form (Fig. 7, *J*–*L* and Movie S5). Our interpretation is that the open state prevents protein-protein interactions when not bound by ATP.

To determine the post-catalysis product state of the kinase catalytic cycle, we utilized ADP as a ligand to determine the ADP-metal-bound FakA structure to 1.25 Å resolution (Fig. S6). The structure of ADP-FakA_N was nearly identical to AMP-PNP-FakA_N aside from the absence of the γ-phosphate as superposition yielded an RMSD of 0.178 Å (Fig. S6). ADP is likely then released to return FakA to the apo state after the fatty acid is phosphorylated. Our crystallography data provides snapshots of the structural changes that occur in the FakA active site following the binding of ATP during the kinase catalytic cycle.

## Discussion

While the FAK system’s importance in the use of exoFAs for lipid synthesis is known, little is known about the mechanism by which fatty acids are phosphorylated by the system or the identity of the composition of the complex. Our data, along with others, demonstrate that the FakA protein contains three specific domains: a catalytic N-terminal domain that phosphorylates a fatty acid, a Middle domain involved in dimerization, and a C-terminal domain with structural similarity to acyl-binding proteins like FakBs. We have provided context to kinase function during catalysis through a comparison of our newly solved Apo-FakA_N and AMP-PNP-FakA_N structures (Fig. S7). In particular, the α1-2 and α5-6 loops move to bind and secure ATP in preparation for the fatty acid-bound FakB binding in a way to position the γ-phosphate in proximity to the fatty acid head group. Of note, both loops are highly conserved among FakA homologs in Gram-positive bacteria (Fig. 7, blue regions). We have termed the α1-2 loop an activating loop since it (1) contains critical Asp residues that must reposition upon ATP binding for kinase activity, (2) stabilizes that ATP molecule, and (3) would sterically hinder access by other proteins to the active site if it did not collapse around the ATP. We predict that this region of FakA could provide a regulatory mechanism to allow FAK complex formation only when ATP is bound. Indeed, the collapse of the activating loop changes the face of FakA, exposing an altered interface containing conserved hydrophobic residues that we predict facilitate complex formation (Figs. S5 and S7).

We identified a metal binding pocket within the Middle domain of FakA that is necessary for kinase function and FakA’s downstream effects on virulence and growth (Figs. 4 and 5). We provide evidence that this pocket is involved in the maintenance of the structure necessary for FakA dimerization (Fig. 3). Our work identifies and supports specific amino acids within this region (C240, H282, and H284) that are essential for kinase function. This metal binding is indeed important for function in the bacteria since we show that downstream *in vivo* effects on α-hemolysin and resistance to toxic UFAs are abrogated when this pocket is mutated to alanines (Fig. 5). These residues are 100% conserved among FakA homologs that we have examined (Fig. S7). It is unlikely that these residues would comprise part of the active site as previously suggested (30). Instead, we predict that the Zn ion stabilizes the β-sheets of the Middle domain, allowing proper folding. Based on the importance of zinc, it is tempting to speculate that there could also be a link between metal homeostasis and fatty acid metabolism. We show that FakA primarily homodimerizes in solution and our data supports the model that the Middle domain mediates this interaction (Fig. 3). We see two possible scenarios for the importance of this dimer. The first is that the FakA dimer is the “off state” and interaction with FakB occurs as a heterodimer following dissolution of the FakA dimer. Thus, FakA dimerization could be a regulatory mechanism. A more likely model is that FakA remains as a dimer during complex formation with FakB to form a heterotetramer. Dimerization of FakA may serve as an anchor for the complex and structurally support the binding of FakA and FakB. This would be particularly important in the transphosphorylation model predicted by AlphaFold (Fig. 1*B*). In this model, dimerization would be necessary for an active complex since the N-terminal domain of one FakA monomer interacts with FakB which is engaged with the C-terminal domain of the other FakA monomer. This could only happen if FakA remained as a dimer as indicated by our BLI data. Determining if FakA remains a dimer in the active FAK complex and whether the dimerization state serves a regulatory function will be one focus of future studies.

The studies by us and others provide the first insights into the structures of these ubiquitous proteins (30, 31); however, the details of how the phosphate is transferred from ATP to the fatty acid are yet to be elucidated. Our structures indicate that the activating loop movement alters the face of FakA right at the ATP γ-phosphate that will be transferred to the fatty acid, which facilitates access to the active site. However, the nature of the active site and what protein interacts with FakA-N remains uncertain. FakB1 and FakB2 possess a channel in which the hydrophobic tail of the fatty acid is positioned, leaving the headgroup exposed (26). In one model (30), the FakA kinase domain and FakB form an active site to allow phosphotransfer of the ATP γ-phosphate in FakA to the fatty acid headgroup exposed by FakB. This model is unaffected by whether the complex exists as a heterodimer or heterotetramer (Fig. 1). In this model, the FakA C-terminal domain serves as a support for FakB, binding to the opposite side of FakB compared to the position of the fatty acid. This specific interaction provides additional stabilization to the interaction between FakA and FakB. Indeed, the C-terminal amino acid of FakA is important for interacting with FakB which we hypothesize stabilizes the complex (30). A second model was recently proposed based on data using the *S. suis* Fak proteins (31). In this model, FakB binds the C-terminal domain of FakA, causing a conformational change to release the fatty acid into a pocket formed by FakA_C and FakA_N from another FakA molecule for phosphorylation. The model in which FakB is only bound to FakA_C may be an intermediate state of the complex before it reaches the active state. Interestingly, the affinity for FakB1 for FakA_C is lower than that for full-length FakA indicating that more than just contact with FakA_C facilitates complex formation (30). Distinguishing between these two models and obtaining the structure of a functional complex will be the focus of our future studies. However, regardless of whether FakA-N interacts with FakA_C or FakB to form the active site, our data provide new insights into changes that occur to accommodate this interaction. Before ATP binding, FakA_N exists in an open state with a potential steric gate to prevent aberrant protein interactions. However, upon ATP binding, the gate collapses and the face of Fak_N changes to accommodate its interacting partner. These changes would be necessary to present the fatty acid for phosphorylation.

*S. aureus* is a prevalent multi-drug-resistant pathogen. One of our long-term goals is to use fatty acid metabolism as a potential target for therapeutic development. Indeed, FASII inhibitors are one drug class that has been suggested to serve as novel therapeutics with one of the most well-known being Triclosan. However, resistance to FASII inhibitors is known and Gram-positive bacteria could bypass them *via* exoFA acquisition through the FAK complex. Thus, finding potential targets within the FAK complex is necessary to develop therapeutic inhibitors to be used in combination with FASII inhibitors, effectively shutting down any mechanism of providing fatty acids for lipid synthesis. A better understanding of the FakA-FakB complex structure and functional dynamics is necessary to comprehend the mechanism of exoFA utilization in Gram-positive bacteria. Our data as well as others are making progress toward this goal, however, much is left to be elucidated. Further studies of the active complex as well as more knowledge of the regulatory mechanisms of this protein are necessary. Elucidation of this FAK complex will provide insight into a conserved mechanism among Gram-positives as well as inform downstream medicinal chemistry and molecular docking to create effective therapeutics and combat antibiotic resistance.

## Experimental procedures

### Bacterial strains, media, growth conditions, and cloning

The strains and plasmids used in this study are provided in Table S4. *S. aureus* strains were grown in tryptic soy broth or agar (TSB or TSA) supplemented with chloramphenicol (10 μg·ml^−1^) or erythromycin (5 μg·ml^−1^) when necessary. *Escherichia coli* strains were grown in lysogeny broth (LB) or 2X-YT medium with ampicillin (100 μg·ml^−1^) or kanamycin (50 μg·ml^−1^) when necessary. PCR reactions were performed with KOD Polymerase (Novagen), and products were cleaned using the ZymoResearch Clean and Concentrator kit between steps. All inserts were sequenced at ACGT, Inc to ensure no unintended changes occurred. Oligonucleotides are provided in Table S5.

### Construction of FakA variant expression plasmids for *S. aureus*

A PCR splicing by overlap extension (SOEing) technique was used to construct a *fakA*^*C240A*^ variant. PCR amplification using oligos JBHEM1, JBKU81, JBKU82 and CNK26 were performed using pCK13 as a template. The products from those reactions were used in a SOEing PCR with JBHEM1 and CNK26. The final PCR product was digested with BamHI and PstI and cloned into the same sites of pCM28 to create pJB1036. A similar process was used for the *fakA*^*H282A, H284A*^ variant. The first two PCR products used primer pairs JBHEM1/MP11 or CNK26/MP7 were mixed and used in a SOEing PCR, with subsequent cloning into pCM28 digested with BamHI and PstI to generate pMP4.

### Construction of expression plasmids for recombinant protein purification

The gene encoding FakA^D38A, D40A^ was amplified from pCK2 by PCR using primers CNK24 and CNK25 and cloned into pET28a digested with Nde1 and Xho1 such that an N-terminal His-tag was added to produce pCK10. For His-FakB2, the *fakB2* gene was amplified from AH1263 chromosome DNA using primers CNK39 and CNK40 and cloned into the NdeI and XhoI sites of pET28a to generate pCK23. The constructs containing the FakA Middle and C-terminal domains were amplified by PCR using primers JBKU104 and JB42 with pJB165 as the template and similarly cloned into pET28a to produce pJB1042. The constructs containing just the FakA C-terminal domain (pMJM2-4) as well as GST-TEV-tagged FakA WT (pMJM2), *fakA*^*H282A, H284A*^ (pMJM3) and *fakA*^*C240A*^ (pMJM4) in pET42A (+) were ordered from Genscript.

### Kinase activity assay

The Universal Kinase Kit (R and D systems. Cat #: EA004) was used following adjusted manufacturer instructions. The substrate mix was prepared in wells of a 96-well plate in a minimum of duplicate by combining 10 μl of ATP (1 mM) (or ADP for positive control) with 15 μl oleic acid (31.4 mM in MeOH) in each well. The enzyme mix was prepared by combining 10 μl of coupling phosphatase (5 ng·μl^−1^), with 7.5 μl FakA (8 μM) and 7.5 μl FakB2 (16.14 μM). The enzyme and substrate mixes were combined in each well and the reaction was incubated at room temperature for 30 min with a control well of only Assay Buffer. 30 μl Malachite Green Reagent A, 100 μl diH_2_O, and 30 μl Malachite Green Reagent B were subsequently added, tapped to mix, then incubated at room temperature for 20 min. A_620_ was measured using a Tecan Spark 10 M plate reader to determine phosphate production. This assay tracks inorganic phosphate levels. In short, the kinase activity of the Fak complex converts ATP to ADP which is subsequently converted to AMP and inorganic phosphate by a coupling phosphatase. It is the inorganic phosphate that interacts with malachite green for the colorimetric change measured at 620 nm. In all assays, an ADP control was used as a control for assay function.

### Inductively coupled plasma mass spectrometry (ICP-MS)

Purified proteins were diluted to a concentration of 1 mg·mL^−1^ in Gel filtration buffer (50 mM Tris HCl [pH 7.4], 150 mM KCl, 1 mM TCEP, 5% glycerol in diH_2_O). Protein samples were compared to control obtained as the flow-through of buffer through a 10 kDa concentrator during sample preparation and termed buffer control. Samples were provided to the K-State Veterinary Diagnostic Laboratory and a Trace Mineral Panel was performed using a PerkinElmer NexIon 350 ICP-MS instrument.

### Hemolysis assay

Hemolysis assays were performed as previously described (9). Briefly, overnight cultures were diluted to an OD600 = 1, and 1 μl was spotted on TSA with 5% rabbit blood. Hemolysis activity was observed after 48 h of incubation at 37 °C.

### Bio-layer interferometry (BLI) for FakA-FakB2 interaction

BLI was performed on the Octet RED96e in the Kansas Intellectual and Developmental Disabilities Research Center at the University of Kansas Medical Center. Ni-NTA biosensor tips (Sartorius) were hydrated in Assay Buffer (50 mM Tris HCl [pH 7.4], 150 mM KCl, 1 mM TCEP, 5% glycerol, 10 mM MgCl_2_) for 10 min at room temperature. A baseline measurement was taken in a buffer for 1 min followed by loading/immobilization of 2 μg·mL^−1^ purified His-tagged FakB2 protein for 4 min. The tips were then blocked with 0.1 mg·mL^−1^ Bovine Serum Albumin (BSA) for 2 min followed by a 2-min wash in Assay Buffer. A second baseline measurement was taken in Assay Buffer for 1 min. Varying concentrations of untagged FakA purified protein were then allowed to bind to the immobilized FakB2 for 5 min followed by a 10-min dissociation period in Assay Buffer. Data were used to determine an approximate K_D_. In additional studies, FakB was loaded at a range of 2 to 20 μg·mL^−1^ yielding a K_D_ range of 4.34 to 15 nM, with a mean value of 10.6 nM.

### Mass photometry (MP)

MP experiments were performed on a Refeyn TwoMP (Refeyn Ltd) at the University of Iowa Protein & Crystallography Facility. Microscope coverslips (No. 1.5H, 24 mm × 50 mm, Thorlabs Inc) were cleaned by serial rinsing with Milli-Q water and HPLC-grade isopropanol (Sigma Aldrich), on which a CultureWell silicone gasket (Grace Bio-labs) was then placed. All MP measurements were performed at room temperature in buffer (50 mM Tris [pH 7.4], 150 mM KCl, 1 mM TCEP, 10 μM ZnSO_4_). For each measurement, 10 μl of buffer was placed in the well for focusing, after which 10 μl of 20 nM protein was introduced, for a final concentration of 10 nM. Movies were recorded for 60 s at 50 fps under standard settings. MP measurements were calibrated using an in-house prepared protein standard mixture: β-Amylase (56,112, and 224 kDa), and Thyroglobulin (670 kDa). MP data were processed using DiscoverMP (Refeyn Ltd).

### Protein purification and structural analysis

Full details are provided in the Supporting Information.

## Data availability

The coordinates and structure factors for Apo-FakA_N (8VIR), ADP-FakA_N (8VIT), Mn-FakA_N (8VIP), and AMP-PNP-FakA_N (8VIQ) have been deposited to the Worldwide Protein Databank (wwPDB). The SAXS data for full-length FakA have been deposited in the Small Angle Scattering Biological Data Bank (SASBDB) under the accession code SASDTW8.

## Supporting information

This article contains supporting information (33, 34, 35, 36, 37, 38, 39, 40, 41, 42, 43, 44, 45, 46, 47, 48, 49, 50, 51, 52, 53, 54, 55, 56, 57, 58, 59, 60, 61, 62, 63, 64, 65, 66, 67).

## Conflict of interest

The authors declare that they have no conflicts of interest with the contents of this article.

## References

[1] Wertheim H.F., Melles D.C., Vos M.C., van Leeuwen W., van Belkum A., Verbrugh H.A. (2005). The role of nasal carriage in *Staphylococcus aureus* infections. Lancet Infect. Dis..

[2] Sakr A., Bregeon F., Mege J.L., Rolain J.M., Blin O. (2018). *Staphylococcus aureus* nasal colonization: an update on mechanisms, epidemiology, risk factors, and subsequent infections. Front. Microbiol..

[3] Tong S.Y., Davis J.S., Eichenberger E., Holland T.L., Fowler V.G. (2015). *Staphylococcus aureus* infections: epidemiology, pathophysiology, clinical manifestations, and management. Clin. Microbiol. Rev..

[4] Klevens R.M. (2007). Invasive methicillin-resistant *Staphylococcus aureus* infections in the United States. JAMA.

[5] Knox J., Uhlemann A.C., Lowy F.D. (2015). *Staphylococcus aureus* infections: transmission within households and the community. Trends Microbiol..

[6] Lee B.Y., Singh A., David M.Z., Bartsch S.M., Slayton R.B., Huang S.S. (2013). The economic burden of community-associated methicillin-resistant *Staphylococcus aureus* (CA-MRSA). Clin. Microbiol. Infect..

[7] GBD 2019 Antimicrobial Resistance Collaborators (2022). Global mortality associated with 33 bacterial pathogens in 2019: a systematic analysis for the Global Burden of Disease Study 2019. Lancet.

[8] Vestergaard M., Frees D., Ingmer H. (2019). Antibiotic resistance and the MRSA problem. Microbiol. Spectr..

[9] Bose J.L., Daly S.M., Hall P.R., Bayles K.W. (2014). Identification of the *Staphylococcus aureus* vfrAB operon, a novel virulence factor regulatory locus. Infect. Immun..

[10] Parsons J.B., Broussard T.C., Bose J.L., Rosch J.W., Jackson P., Subramanian C. (2014). Identification of a two-component fatty acid kinase responsible for host fatty acid incorporation by *Staphylococcus aureus*. Proc. Natl. Acad. Sci. U. S. A..

[11] Krute C.N., Rice K.C., Bose J.L. (2017). VfrB is a key activator of the *Staphylococcus aureus* SaeRS two-component system. J. Bacteriol..

[12] Ericson M.E., Subramanian C., Frank M.W., Rock C.O. (2017). Role of fatty acid kinase in cellular lipid homeostasis and SaeRS-dependent virulence factor expression in *Staphylococcus aureus*. MBio.

[13] Krute C.N., Ridder M.J., Seawell N.A., Bose J.L. (2019). Inactivation of the exogenous fatty acid utilization pathway leads to increased resistance to unsaturated fatty acids in *Staphylococcus aureus*. Microbiology (Reading).

[14] Frank M.W., Yao J., Batte J.L., Gullett J.M., Subramanian C., Rosch J.W. (2020). Host fatty acid utilization by *Staphylococcus aureus* at the infection site. mBio.

[15] Lopez M.S., Tan I.S., Yan D., Kang J., McCreary M., Modrusan Z. (2017). Host-derived fatty acids activate type VII secretion in *Staphylococcus aureus*. Proc. Natl. Acad. Sci. U. S. A..

[16] Ridder M.J., Daly S.M., Triplett K.D., Seawell N.A., Hall P.R., Bose J.L. (2020). *Staphylococcus aureus* fatty acid kinase FakA modulates pathogenesis during skin infection *via* proteases. Infect. Immun..

[17] Demars Z., Bose J.L. (2018). Redirection of metabolism in response to fatty acid kinase in *Staphylococcus aureus*. J. Bacteriol..

[18] Fischer C.L., Blanchette D.R., Brogden K.A., Dawson D.V., Drake D.R., Hill J.R. (2014). The roles of cutaneous lipids in host defense. Biochim. Biophys. Acta.

[19] Wertz P.W., de Szalay S. (2020). Innate antimicrobial defense of skin and oral mucosa. Antibiotics (Basel).

[20] Nguyen M.T., Hanzelmann D., Hartner T., Peschel A., Gotz F. (2016). Skin-specific unsaturated fatty acids boost the *Staphylococcus aureus* innate immune response. Infect. Immun..

[21] Delekta P.C., Shook J.C., Lydic T.A., Mulks M.H., Hammer N.D. (2018). *Staphylococcus aureus* utilizes host-derived lipoprotein particles as sources of fatty acids. J. Bacteriol..

[22] Morvan C., Halpern D., Kenanian G., Hays C., Anba-Mondoloni J., Brinster S. (2016). Environmental fatty acids enable emergence of infectious *Staphylococcus aureus* resistant to FASII-targeted antimicrobials. Nat. Commun..

[23] Morvan C., Halpern D., Kenanian G., Pathania A., Anba-Mondoloni J., Lamberet G. (2017). The *Staphylococcus aureus* FASII bypass escape route from FASII inhibitors. Biochimie.

[24] Wallace J., Bowlin N.O., Mills D.M., Saenkham P., Kwasny S.M., Opperman T.J. (2015). Discovery of bacterial fatty acid synthase type II inhibitors using a novel cellular bioluminescent reporter assay. Antimicrob. Agents Chemother..

[25] Parsons J.B., Frank M.W., Jackson P., Subramanian C., Rock C.O. (2014). Incorporation of extracellular fatty acids by a fatty acid kinase-dependent pathway in *Staphylococcus aureus*. Mol. Microbiol..

[26] Gullett J.M., Cuypers M.G., Grace C.R., Pant S., Subramanian C., Tajkhorshid E. (2022). Identification of structural transitions in bacterial fatty acid binding proteins that permit ligand entry and exit at membranes. J. Biol. Chem..

[27] Cuypers M.G., Subramanian C., Gullett J.M., Frank M.W., White S.W., Rock C.O. (2019). Acyl-chain selectivity and physiological roles of *Staphylococcus aureus* fatty acid-binding proteins. J. Biol. Chem..

[28] Parsons J.B., Frank M.W., Subramanian C., Saenkham P., Rock C.O. (2011). Metabolic basis for the differential susceptibility of Gram-positive pathogens to fatty acid synthesis inhibitors. Proc. Natl. Acad. Sci. U. S. A..

[29] DeMars Z., Singh V.K., Bose J.L. (2020). Exogenous fatty acids remodel *Staphylococcus aureus* lipid composition through fatty acid kinase. J. Bacteriol..

[30] Subramanian C., Cuypers M.G., Radka C.D., White S.W., Rock C.O. (2022). Domain architecture and catalysis of the *Staphylococcus aureus* fatty acid kinase. J. Biol. Chem..

[31] Shi Y., Zang N., Lou N., Xu Y., Sun J., Huang M. (2022). Structure and mechanism for streptococcal fatty acid kinase (Fak) system dedicated to host fatty acid scavenging. Sci. Adv..

[32] Jumper J., Evans R., Pritzel A., Green T., Figurnov M., Ronneberger O. (2021). Highly accurate protein structure prediction with AlphaFold. Nature.

[33] Kabsch W. (1988). Automatic indexing of rotation diffraction patterns. J. Appl. Crystallogr..

[34] Kabsch W. (2010). XDS. Acta Crystallogr. D Biol. Crystallogr..

[35] Vonrhein C., Flensburg C., Keller P., Sharff A., Smart O., Paciorek W. (2011). Data processing and analysis with the autoPROC toolbox. Acta Crystallogr. D Biol. Crystallogr..

[36] Evans P.R. (2011). An introduction to data reduction: space-group determination, scaling and intensity statistics. Acta Crystallogr. D Biol. Crystallogr..

[37] Skubak P., Pannu N.S. (2013). Automatic protein structure solution from weak X-ray data. Nat. Commun..

[38] Sheldrick G.M. (2010). Experimental phasing with SHELXC/D/E: combining chain tracing with density modification. Acta Crystallogr. D Biol. Crystallogr..

[39] Murshudov G.N., Vagin A.A., Dodson E.J. (1997). Refinement of macromolecular structures by the maximum-likelihood method. Acta Crystallogr. D Biol. Crystallogr..

[40] Abrahams J.P., Leslie A.G.W. (1996). Methods used in the structure determination of bovine mitochondrial F_1_ ATPase. Acta Crystallogr. Section D.

[41] Zhang K.Y.J., Cowtan K., Main P. (1997). Methods in Enzymology.

[42] Cowtan K. (2006). The Buccaneer software for automated model building. 1. Tracing protein chains. Acta Crystallogr. D Biol. Crystallogr..

[43] Winn M.D., Ballard C.C., Cowtan K.D., Dodson E.J., Emsley P., Evans P.R. (2011). Overview of the CCP4 suite and current developments. Acta Crystallogr. D Biol. Crystallogr..

[44] Adams P.D., Afonine P.V., Bunkoczi G., Chen V.B., Davis I.W., Echols N. (2010). PHENIX: a comprehensive Python-based system for macromolecular structure solution. Acta Crystallogr. D Biol. Crystallogr..

[45] Emsley P., Lohkamp B., Scott W.G., Cowtan K. (2010). Features and development of coot. Acta Crystallogr. D Biol. Crystallogr..

[46] McCoy A.J., Grosse-Kunstleve R.W., Adams P.D., Winn M.D., Storoni L.C., Read R.J. (2007). *Phaser* crystallographic software. J. Appl. Crystallogr..

[47] Chen V.B., Arendall W.B., Headd J.J., Keedy D.A., Immormino R.M., Kapral G.J. (2010). MolProbity: all-atom structure validation for macromolecular crystallography. Acta Crystallogr. D Biol. Crystallogr..

[48] Potterton L., McNicholas S., Krissinel E., Gruber J., Cowtan K., Emsley P. (2004). Developments in the CCP4 molecular-graphics project. Acta Crystallogr. D Biol. Crystallogr..

[49] Krissinel E. (2012). Enhanced fold recognition using efficient short fragment clustering. J. Mol. Biochem..

[50] Kirby N., Cowieson N., Hawley A.M., Mudie S.T., McGillivray D.J., Kusel M. (2016). Improved radiation dose efficiency in solution SAXS using a sheath flow sample environment. Acta Crystallogr. D Struct. Biol..

[51] Hopkins J.B., Gillilan R.E., Skou S. (2017). BioXTAS RAW: improvements to a free open-source program for small-angle X-ray scattering data reduction and analysis. J. Appl. Crystallogr..

[52] Meisburger S.P., Case D.A., Ando N. (2020). Diffuse X-ray scattering from correlated motions in a protein crystal. Nat. Commun..

[53] Svergun D. (1992). Determination of the regularization parameter in indirect-transform methods using perceptual criteria. J. Appl. Crystallogr..

[54] Franke D., Svergun D.I. (2009). DAMMIF, a program for rapid ab-initio shape determination in small-angle scattering. J. Appl. Crystallogr..

[55] Volkov V.V., Svergun D.I. (2003). Uniqueness of ab initio shape determination in small-angle scattering. J. Appl. Crystallogr..

[56] Svergun D.I. (1999). Restoring low resolution structure of biological macromolecules from solution scattering using simulated annealing. Biophys. J..

[57] Grant T.D. (2018). Ab initio electron density determination directly from solution scattering data. Nat. Methods.

[58] Pelikan M., Hura G.L., Hammel M. (2009). Structure and flexibility within proteins as identified through small angle X-ray scattering. Gen. Physiol. Biophys..

[59] Pettersen E.F., Goddard T.D., Huang C.C., Couch G.S., Greenblatt D.M., Meng E.C. (2004). UCSF Chimera--a visualization system for exploratory research and analysis. J. Comput. Chem..

[60] Boles B.R., Thoendel M., Roth A.J., Horswill A.R. (2010). Identification of genes involved in polysaccharide-independent *Staphylococcus aureus* biofilm formation. PLoS One.

[61] Kreiswirth B.N., Löfdahl S., Betley M.J., O’Reilly M., Schlievert P.M., Bergdoll M.S. (1983). The toxic shock syndrome exotoxin structural gene is not detectably transmitted by a prophage. Nature.

[62] Pang Y.Y., Schwartz J., Thoendel M., Ackermann L.W., Horswill A.R., Nauseef W.M. (2010). agr-Dependent interactions of *Staphylococcus aureus* USA300 with human polymorphonuclear neutrophils. J. Innate Immun..

[63] Evans P. (2006). Scaling and assessment of data quality. Acta Crystallogr. D Biol. Crystallogr..

[64] Diederichs K., Karplus P.A. (1997). Improved R-factors for diffraction data analysis in macromolecular crystallography. Nat. Struct. Biol..

[65] Weiss M.S. (2001). Global indicators of X-ray data quality. J. Appl. Crystallogr..

[66] Karplus P.A., Diederichs K. (2012). Linking crystallographic model and data quality. Science.

[67] Evans P. (2012). Biochemistry. Resolving some old problems in protein crystallography. Science.

